# Fetal hemoglobin enables malaria parasite growth in sickle cells but augments production of transmission stage parasites

**DOI:** 10.1371/journal.pone.0325797

**Published:** 2025-07-08

**Authors:** Catherine Lavazec, Cheikh Loucoubar, Florian Dupuy, Jean-François Bureau, Isabelle Casadémont, Bronner Gonçalves, Swee Lay Thein, Mark Lathrop, Sandrine Laurance, Camille Roussel, Caroline Le Van Kim, Yves Colin, Mariane De Montalembert, Anavaj Sakuntabhai, Richard E. Paul

**Affiliations:** 1 Université Paris Cité, Inserm U1016, CNRS UMR, Institut Cochin, Paris, France; 2 Epidemiology, Clinical Research and Data Science Unit, Institut Pasteur of Dakar, Dakar, Senegal; 3 Institut Pasteur, Université Paris Cité, CNRS UMR2000, Ecology and Emergence of Arthropod-borne Pathogens Unit, Paris France; 4 Department of Comparative Biomedical Sciences, School of Veterinary Medicine, University of Surrey,; 5 Sickle Cell Branch, National Heart, Lung and Blood Institutes/ National Institutes of Health, Bethesda, Maryland, United States of America; 6 Victor Philip Dahdaleh Institute of Genomic Medicine at McGill University, Canada; 7 Université Paris Cité, UMR_S1134, Biologie Intégrée du Globule Rouge BIGR, INSERM, Paris, France; 8 Laboratoire d’hématologie générale, Hôpital Necker-Enfants malades, Assistance Publique-Hôpitaux de Paris, Paris, France; 9 Université Paris Cité, Department of General Pediatrics and Pediatric Infectious Diseases, Reference Center for Sickle Cell Disease, Hôpital Necker-Enfants malades, Assistance Publique-Hôpitaux de Paris, Paris, France; Penn State Health Milton S Hershey Medical Center, UNITED STATES OF AMERICA

## Abstract

Sickle cell trait is the quintessential example of the human evolutionary response to malaria, providing protection against severe disease, but leading to sickle cell disease (SCD) in the homozygous state. Fetal Hemoglobin (HbF) reduces the pathology of SCD and several mutations lead to the prolonged production of HbF into childhood and adult life. HbF has been suggested to contribute to protection against malaria. Two long-term cohorts were genotyped for three quantitative trait loci associated with HbF production and analyzed for HbF titers, malaria clinical episodes and the production of parasite stages infectious to mosquitoes, gametocytes, in asymptomatic infections. *Plasmodium falciparum* parasites were also grown *in vitro* in HbSS cells with measured levels of HbF. The genetic determinants of prolonged HbF production were associated with increased HbF titers and that increased HbF afforded protection from malaria disease but increased the production of gametocytes. The presence of HbF in sickled red cells was also shown in *in vitro* culture to enable parasite persistence in conditions otherwise deleterious for the parasite and enabled complete maturation of gametocytes. The beneficial personal effect of HbF, whether through protection against malaria or alleviating effects of SCD, is seemingly offset by increased parasite transmissibility and potential disease burden for the community. These individuals represent a potentially important reservoir of infection and could be targeted in elimination strategies.

## Introduction

Transmission of the parasite from man to mosquito requires the presence of gametocyte stage parasites. Whilst there have been several epidemiological studies identifying risk factors for the presence of gametocytes, thereby offering a potential target group for treatment, none have been considered sufficiently robust [1–3]. One notable feature was that the production of these gametocyte stages was found to have a strong human genetic basis (heritability), especially at sub-microscopic densities, but only during symptom-free infections [4]. Mutations of the Beta-hemoglobin gene (HBB) have been shown to be associated with gametocyte carriage and transmission to mosquitoes, but explained little of this heritability [2,4,5].

As encapsulated by the Red Queen hypothesis, hosts and pathogens are locked in a co-evolutionary arms race with adaptation and counter-adaptation to promote survival [6]. The human genome has been thus remodeled over the millennia and hemoglobinopathies are quintessential examples of this positive selection, protecting from severe malaria [7,8]. In turn, malaria parasites have been shown to be particularly efficient in responding to host defenses to ensure both their survival, through antigenic variation, and their transmission, through modulation of the sex ratio of the gametocytes, responsible for transmission to mosquitoes, thereby responding to the immediate human immunological and hematological response to infection [9,10].

Hereditary persistence of fetal hemoglobin (HPFH) is a benign condition where the production of fetal hemoglobin (HbF) persists into adulthood and alleviates the severity of sickle cell disease and β-thalassemias [11]. The protective effect afforded by these hemoglobinopathies against malaria has been suggested to be in part due to HbF [12–14]. and gametocytes have been shown *in vitro* to be able to grow in fetal RBCs when added to cultures of normal HbA RBCs [15].

### Evidence before this study

Sickle cell trait of red blood cells has evolved to afford protection against severe malaria. However, in the homozygous form, this results in sickle cell disease (SCD), a life-impeding pathology. The prolonged production of fetal hemoglobin (HbF) can alleviate the pathological consequences of SCD and has also been thought to also afford protection against malaria. To transmit from humans to mosquitoes, the malaria parasite must produce specialized transmission stages, gametocytes. Previous work has shown a strong human genetic basis to gametocyte production, but only in symptom-free malaria infections. We hypothesized that HbF was contributing to gametocyte production. We searched PubMed for articles with the following keywords: Foetal haemoglobin OR Fetal hemoglobin AND Malaria and then Foetal haemoglobin OR Fetal hemoglobin AND Gametocytes. Whilst there were 191 references for the search with malaria, there was only one for the search with gametocytes, reference 15. This article was primarily focused on *in vitro* culture of gametocytes and only cursorily noted that there was development of gametocytes in fetal red blood cells (RBC) added to cultures, but which provided a less favorable environment than those with HbA RBC for growth of the parasites. No studies since this 1978 publication have addressed gametocyte growth in fetal RBC nor has there been any human cohort studies.

Overall, this study aims to address the role that HbF plays not only in protecting from malaria but also in eliciting the parasite response to such an HbF rich environment in two long-term human cohorts in malaria endemic settings and the impact of HbF in sickle cells *in vitro*. Specifically the aims are to address: (1) the association of the three Quantitative Trait loci (QTL) previously shown to induce HbF production with HbF titers in the two study cohorts; (2) the association of these QTL with the number of clinical malaria episodes; (3) the association of these QTLs with gametocyte positivity in asymptomatic infections and (4) the impact of the presence of HbF in HbSS (sickle cells) RBCs in *in vitro* culture.

### Added value of this study

We conducted a human genetic analysis of three quantitative trait loci known to be associated with HbF production in two very long-term cohorts followed for malaria parasite infection and clinical episodes. We found that individuals with mutations associated with HbF production had a reduced number of clinical episodes but produced more gametocytes during asymptomatic infections. We additionally showed in *in vitro* culture that the presence of HbF in RBC enables parasite growth and gametocyte production at tissue level partial pressures of oxygen at which normally parasite lysis would occur.

## Materials and methods

### Study populations, malaria clinical episodes, asymptomatic gametocyte carriage and fetal hemoglobin levels

This study used retrospective malaria data concerning clinical malaria episodes and gametocyte carriage during asymptomatic infections from two long term cohorts, Dielmo and Ndiop as previously described [4,16–19]. Clinical case detection was both active and passive. The gametocyte carriage rate come from the monthly systematic blood slides. The malaria data were collected from 1990–1999 and 1993–1999 in Dielmo and Ndiop respectively. The annual population demography over this period is shown in Table 1A and 1B. Previous typing with microsatellites enabled the construction of a pedigree based on Identity-by-Descent using MERLIN [19,20]. The total pedigrees, in Dielmo and Ndiop respectively, comprised 828 and 948 individuals composed of 206 and 222 nuclear families. For the present study, genotyping for at least one successful single nucleotide polymorphisms (SNP) was achieved for 408 of the 429 individuals in Dielmo for whom malaria phenotypes were available and 662 of the 667 individuals in Ndiop (See Data set). The malaria data used are the residual unexplained variation in the number of clinical *P. falciparum* episodes and the asymptomatic gametocyte carriage rate after accounting for known confounding factors such as age, season and year of study from previous publications [4,19]. These data concern 386 individuals for clinical episodes in Dielmo and 455 individuals in Ndiop. For asymptomatic gametocyte carriage, there were data from 210 individuals in Dielmo and 263 individuals in Ndiop. The HbF proportion of total hemoglobin was measured by high pressure liquid chromatography (HPLC) from the intra-venous samples reported in Sakuntabhai et al. [19].

**Table 1:** Annual demographic age structure (number of individuals) of the two study cohorts.

A. Dielmo										
Age (years)	1990	1991	1992	1993	1994	1995	1996	1997	1998	1999
**0–4**	61	58	57	68	65	67	72	71	74	67
**5–9**	40	47	47	47	50	53	63	58	68	66
**10–14**	29	31	33	35	40	37	44	46	53	54
**15–19**	25	34	26	35	29	30	33	33	41	48
**20–24**	21	26	27	26	27	26	38	26	41	36
**25–29**	21	20	20	18	24	15	21	22	34	37
**30–34**	15	13	13	18	24	22	21	19	19	20
**35–39**	17	15	18	15	14	14	15	13	24	26
**40–44**	6	7	11	10	12	16	16	16	16	15
**45–49**	11	11	11	9	8	8	10	11	12	14
**50–54**	13	9	10	10	11	13	13	12	10	8
**55–59**	4	5	5	7	8	10	11	11	14	15
**60–64**	7	5	7	5	4	1	4	5	7	8
**65–69**	4	5	3	5	6	7	6	6	4	3
**70–74**	6	4	3	2	3	4	5	3	5	5
**75–79**	4	4	5	4	3	3	2	3	3	3
**80–84**	3	4	3	2	2	2	1	2	3	3
**85–89**	1	1	2	3	3	3	4	3	2	2
**90–94**	1	1	1	1	1	1	1	2	3	3
**95–99**	1	1	1	1	1	1	1	1	1	1
**B. Ndiop**										
**Age (years)**	**1993**	**1994**	**1995**	**1996**	**1997**	**1998**	**1999**			
**0–4**	87	87	91	79	101	104	89			
**5–9**	75	67	76	80	81	101	85			
**10–14**	40	50	51	53	61	82	68			
**15–19**	45	48	50	44	47	77	74			
**20–24**	38	32	39	38	42	75	60			
**25–29**	25	23	22	25	23	36	33			
**30–34**	32	25	22	22	25	29	23			
**35–39**	18	23	25	22	22	30	27			
**40–44**	12	13	13	10	19	20	20			
**45–49**	9	12	15	11	16	12	13			
**50–54**	11	12	14	9	11	9	12			
**55–59**	7	6	9	7	9	10	8			
**60–64**	2	4	5	6	7	7	5			
**65–69**	6	4	4	3	5	4	3			
**70–74**	3	3	3	3	2	3	3			
**75–79**	2	1	1	1	1	1	1			
**80–84**	1	1	1	1	1	1	1			
**85–89**	1	1	1	1	1	1	1			
**90–94**	1	1	1	1	1	1	1			
**95–99**	1	1	1	1	1	1	1			

### Genotyping

The DNA samples used were those previously extracted as described in Sakuntabhai *et al*. 2008 [19]. The HBB mutation data used were from Lawaly *et al*. 2010 [4]. Three QTL associated with HPFH are found on chromosomes 2, 6 and 11 (S1 Fig and S1 Table) [21,22]. The following SNPs were genotyped to cover the linkage regions in the two QTL on chromosomes 2 & 6, as well as the Xmn-1 mutation on chromosome 11. SNPs were selected based on the minor allele frequencies in Yoruba populations in HapMap. Shown below the SNPs and the Wildtype/Mutation alleles

Chromosome 2: *rs1427407*-G/T, *rs243027*-T/G, *rs6732518*-T/C

Chromosome 6: *rs11759553*-T/A, *rs11154792*-T/C, *rs9399137*-T/C, *rs6904897*-T/G, *rs4895441*-A/G, *rs1320963*-G/A

Chromosome 11: *rs7482144*-C/T-

Their chromosomal positions and genotype frequencies are shown in Table S1.

### TaqMan assay for SNP genotyping

The PCR reaction was carried out in a 5 μl reaction containing 5 μl of PCR solution and 1 μl of DNA 1 ng/1 μl, which was dried at 37°C before adding PCR solution. TaqMan Universal PCR Master Mix was used. PCR reaction mix consisted of 0.5 μl of 10X PCR buffer, 0.5 μl of 50mM MgCl_2_, 0.1 μl of Reference Dye, 0.02 μl of Platinum Taq (5U/μl) (Invitrogen), 0.312 μl of dNTPs 8 mM, 0.125 μl of 40X TaqMan probe, which consists of forward primer, reverse primer, and 2 fluorescence labeled probes, and 3.568 µl of H_2_O. After the ABI PRISM 96 well Optical Reaction plate was covered with ABI PRISM Optical Adhesive Covers, it was incubated at 95°C for 10 min, amplification was carried out for 40 cycles with the following temperature cycling parameters; 92°C for 0.15 min and 60°C for 1 min.

### *In vitro* experiments

#### Red blood cells.

Hemoglobin genotypes were determined as above. The HbF proportion of total hemoglobin was measured by HPLC on a BioRad Variant II system or by flow cytometry with anti-Human Fetal hemoglobin monoclonal antibody APC conjugated (Invitrogen) using a BD Accuri C6 cytometer. Erythrocytes were stored at 4°C and used within one week after collection. Erythrocytes were washed three times in RPMI 1640 before use.

#### Parasite culture.

The *P. falciparum* clonal line B10 and the Pfs16-GFP line expressing GFP under the control of the gametocyte-specific promoter *Pfs16* were cultivated *in vitro* in human erythrocytes using RPMI 1640 medium supplemented with 10% heat inactivated human serum, 100 µM hypoxanthine and 20 µg/ml gentamycin [23,24]. For each experiment, synchronized schizonts obtained via magnetic purification were used to invade HbSS or HbAA erythrocytes at 5% hematocrit. Gametocytogenesis was induced by decreasing culture hematocrit from 5% to 2%, without addition of any agent. Cultures were maintained at 37°C in a gas environment of 1–2% O_2_/ 5% CO_2_/ 93–94% N_2_. Gametocytemia (i.e., % of erythrocytes infected with gametocytes in the total erythrocyte population) and total parasitemia (i.e., % of erythrocytes infected with gametocytes or asexual parasites in the total erythrocyte population) were monitored daily by microscopy analysis of Giemsa-stained blood smears (at least 2000 erythrocytes were counted for each sample) and by flow cytometry analysis using a BD LSRII cytometer after DNA staining with Hoechst 33342 at 1:20000 (Life technologies). Cultures were performed in triplicate in erythrocytes from the same blood donor.

### Detection of parasites in HbF-containing cells by flow cytometry

HbSS or HbAA erythrocytes infected with the Pfs16-GFP line were fixed for 15 min at room temperature in 1X PBS/ 2.7% formaldehyde/ 0.1% glutaraldehyde and then permeabilized with 1X PBS/ 1% Octyl β-D-glucopyranoside (Sigma-Aldrich) for 15 min at room temperature. Cells were washed in 1X PBS, incubated in saturation solution (1X PBS/ 1% BSA/ 2% goat serum) for 15 min at room temperature and then incubated with anti-Human Fetal hemoglobin monoclonal antibody APC conjugated (Invitrogen) at 1:20 in saturation solution for 20 min at room temperature. After two washes in 1X PBS, cells were resuspended in saturation solution and analyzed using a BD Accuri C6 cytometer.

### Detection of parasites in HbF-containing cells by immunofluorescence microscopy

HbSS or HbAA infected with the Pfs16-GFP line were fixed for 15 min at room temperature in 1X PBS/ 1% paraformaldehyde and then permeabilized with 1X PBS/ 0.1% Triton-X100 (Sigma-Aldrich) for 10 min at room temperature. Samples were washed three times in 1X PBS, smeared on glass slides and dried. The non-specific binding sites were saturated for 2 h in 1X PBS/ 2% BSA and the smears were then incubated with anti-Human Fetal hemoglobin monoclonal antibody APC conjugated (Invitrogen) at 1:500 for 1 h 30. After three washes in 1X PBS/ 2% BSA, the smears were incubated with Alexa594 anti-mouse secondary antibodies at 1:2000 (Life Technologies) and Hoechst 33342 at 1:20000. Smears were observed at 100X magnification using a Leica DMi8 fluorescence microscope.

### Statistics

As mentioned above, the data for each malaria-related phenotype had been first analysed using multivariate regression analysis to take into account known confounding factors such as age, season and year of study. The residual unexplained variation in each phenotype was used for further genetic association analysis. For analysis of HbF levels, a Generalized Linear Model (GLM) was fitted taking into account age and parasite positivity. As for the malaria phenotypes, the residuals from this analysis were then used for the genetic association analyses. For the malaria phenotype association population-based multivariable analyses with the SNPs in the three QTL associated with HPFH a Generalized Linear Mixed Model was fitted with the genetic related matrix of all individuals in the cohorts as a factor in the random model as previously described [25,26](S1 File). A Poisson error structure was implemented (thus a loglinear regression). All statistical analyses were performed in Genstat version 22 [27]. Heritability (*h*^*2*^) was evaluated by using the SOLAR software package (version 2.1.4), as described previously [4,28].

### Ethics

This is a retrospective analysis of malaria and related blood parameters from the Dielmo and Ndiop longitudinal cohorts that started in 1990 and 1993 respectively [16,17]. The malaria data used were previously published [4,19]. The HbF data were from samples taken for the aforementioned studies but hitherto unpublished. The project protocol and objectives had been carefully explained to the assembled village populations and informed consent individually obtained from all subjects either by signature or by thumbprint on a voluntary consent form written in both French and in Wolof, the local language. Consent had been obtained in the presence of the school director, an independent witness. For very young children, parents or designated tutors signed on their behalf. The protocol was approved by the Ethical Committee of the Pasteur Institute of Dakar and the Ministry of Health of Senegal. An agreement between Institut Pasteur de Dakar, Institut de Recherche pour le Développement and the Ministère de la Santé et de la Prévention of Senegal defined all research activities in the study cohorts. Each year, the project was re-examined by the Conseil de Perfectionnement de l’Institut Pasteur de Dakar and the assembled village population; informed consent was individually renewed from all subjects. Every person satisfying adhesion conditions could become a volunteer and every volunteer could leave the study at any time, therefore forming a dynamic open cohort. Further details of the study sites and adhesion criteria are previously described [16,29].

## Results

HbF levels from 497 (N = 285 Dielmo, N = 212 Ndiop) symptom-free individuals from two family-based cohorts in a malaria endemic zone in Senegal were measured and found to follow a log-series distribution (S2 Fig), yielding a mean HbF level of 1.58% (variance 1.42%) in Dielmo and 1.39% (variance 1.00%) in Ndiop. HbF levels decreased with age (S3 Fig). Fitting an exponential curve revealed a sharp decrease in HbF levels until a lower asymptote of 1.21% at ~10 years of age in Dielmo, explaining 21% of the variation in HbF levels. The exponential fit was worse (6% variance explained) for Ndiop, with a lower asymptote of 1.12% attained at ~15 years of age.

Of 285 sampled individuals in Dielmo, 64% were blood slide positive for *Plasmodium falciparum* malaria parasites and 5.4% *P. falciparum* and *Plasmodium ovale* mixed infections. Only two *P. falciparum* blood slides had parasite densities in the range of 100−500 parasites/uL, the remainder had densities of less than 100 parasites/uL. All mixed infections had low densities (<100 parasites/uL) and were excluded from further analyses. In Ndiop, of 212 sampled individuals, 35% were *P. falciparum* positive. All parasite densities were less than 100 parasites/uL. Parasite infection was associated with increased HbF levels by 29.7% and 34.3% in Dielmo and Ndiop, respectively (Loglinear regression; P = 0.033 & P = 0.021). After accounting for the effects of age and parasite presence, gender not being found to be associated with HbF levels, the heritability of the residual HbF levels was estimated to be 43.1% (SE 10.9%, P = 4x10^-7^) in Dielmo and 48.7% (SE 16.2%, P = 4.8x10^-4^) in Ndiop.

The Dielmo cohort was genotyped for the sickle mutation (*rs334*) and several SNPs in the three QTL (S1 Table). Only two individuals were HbSS. Using the family-based likelihood ratio test [20], SNPs *rs1427407* on chromosome 2, *rs11759553* on chromosome 6 and the *Xmn1-HBG2* (*rs7482144*) in the HBB cluster were associated with HbF titre (P = 3.2 x 10^−7^, P = 0.0009 and P = 0.017, respectively). There were no significant associations for the other SNPs studied. Population based analyses confirmed the impact of *rs1427407* and *rs11759553*, accounting for 20% and 7% of variation in HbF levels, respectively. The mutation alleles were recessive for all three loci with respect to the HbF phenotype: associated genotypes are thus *rs1427407*-TT, *rs11759553*-AA, *Xmn1-HBG2*-TT. *Xmn1-HBG2* was found to have a significant effect on HbF only when co-occurring with HbS (HbSS and HbAS combined), accounting for 7% of variation in HbF levels.

**Fig 1:**
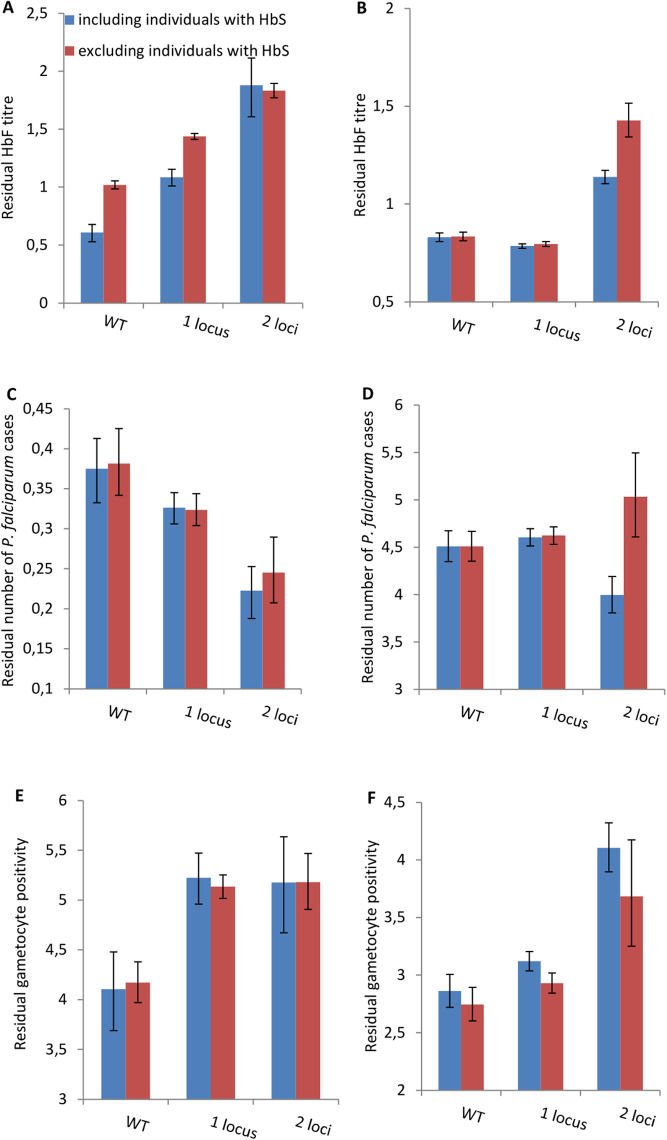
The effect of number of fetal hemoglobin – associated mutant genotypes at one locus and at two or three loci of any of the three SNPs on HbF titre in (A) Dielmo and (B) Ndiop, Number of *P. falciparum* clinical episodes (C) Dielmo and (D) Ndiop and the Gametocyte prevalence rate (E) Dielmo and (F) Ndiop. WT – Wild Type. Shown are the means and standard of errors from the loglinear regression analyses on the residual values for each of three variables (see Supplementary materials). Blue histograms include individuals with sickle cell trait (HbS), red histograms have excluded the HbS individuals prior to the analyses.

To assess whether there was a dose effect with increasing number of mutant genotypes at the three loci, we categorised individuals into the number of mutant genotypes for these three loci. HbF levels increased additively with increasing number of HbF-associated SNPs (N = 181: Mutant genotype at one locus of any of the three SNPs, t = 9.02, P < 0.001; mutant genotype at two or three loci of any of the three SNPs, t = 12.2, P < 0.001)(Fig 1). As shown in Fig 1, analysis with the exclusion of individuals HbS made little difference.

We then examined the association of these three HbF-related SNP recessive mutant genotypes with the number of clinical malaria episodes and the proportion of asymptomatic infections that were positive for gametocytes. In the population-based multivariable association analysis, *rs1427407*-TT and *Xmn1-HBG2*-TT were associated with a decrease in the number of clinical episodes (P = 0.021 and P = 0.018 respectively); *rs11759553*-AA was not found to be associated with the number of clinical episodes (P = 0.278). We then categorised individuals by the number of mutant genotypes of any of the three loci and re-performed the association analyses. The number of clinical episodes decreased by 13% with inheritance of one and 40% with two HbF-related SNPs; there were no individuals with mutant genotypes at all three loci (N = 313, mutant genotype at one locus of any of the three SNPs t = 1.15, P = 0.25; mutant genotype at two loci of any of the three SNPs t = 2.96, P < 0.05). Inheritance of two HbF-related SNPs significantly decreased the number of clinical episodes as compared to inheritance of only 1 SNP (t = 2.50, P < 0.05); there was only one individual with mutant genotypes at all three loci. This protective effect remained after exclusion of any individuals with HbS (N = 283, mutant genotype at one locus of any of the three SNPs t = 1.31, P = 0.19; mutant genotype at two loci of any of the three SNPs, t = 2.22, P < 0.05).

Analysis of gametocyte positivity by population-based analysis revealed an increase in gametocyte positivity for *rs1427407*-TT and *rs11759553*-AA (P = 0.034 and P = 0.007 respectively); *Xmn1-HBG2*-TT was not found to be associated (P = 0.399). Gametocyte positivity increased with inheritance of any HbF-related SNPs by 27% (N = 150: mutant genotype at one locus of any of the three SNPs t = 2.23, P < 0.05; mutant genotype at two loci of any of the three SNPs t = 1.73, P = 0.09); there were no individuals with mutant genotypes at all three loci. For gametocyte prevalence, there was no difference between inheriting one or two HbF-related SNPs (t = 0.09, P = 0.46) The association of gametocyte positivity with inheritance of any HbF-related SNPs became even stronger upon exclusion of any individuals with HbS (N = 137, mutant genotype at one locus of any of the three SNPs, t = 3.85, P < 0.001; mutant genotype at two loci of any of the three SNPs, t = 2.97, P < 0.01) (Fig 1).

We then replicated these findings in Ndiop (N = 667) that had a ten-fold lower level of malaria transmission and composed of a different ethnicity. In the population-based analysis, *rs1427407*-TT, *rs11759553*-AA and *Xmn1-HBG2*-TT were all found to be positively associated with HbF titre (P < 0.001, P = 0.014 and P = 0.037 respectively). HbF was again found to increase additively with increasing number of HbF-related SNPs (N = 124, mutant genotype at one locus of any of the three SNPs, t = 1.84, P = 0.07; mutant genotype at two loci of any of the three SNPs, t = 7.91, P < 0.001); no individuals had mutant genotypes at all three loci for any of the phenotypes studied. In the population-based analysis, only *Xmn1-HBG2*-TT was found to be protective against the number of clinical episodes (P < 0.001). Nevertheless, categorising individuals into the number of mutant genotypes at any of the three loci revealed a decreasing number of clinical malaria episodes with an increasing number of SNPs (N = 389, mutant genotype at one locus of any of the three SNPs, t = 0.51, P = 0.61; mutant genotype at two loci of any of the three SNPs, t = 2.02, P < 0.05). Inheritance of two HbF-related SNPs significantly decreased the number of clinical episodes as compared to inheritance of only one SNP (t = 2.73, P < 0.05). This protective effect was lost after exclusion of any individuals with HbS, potentially because of simultaneous exclusion of *Xmn1-HBG2* (N = 343, mutant genotype at one locus of any of the three SNPs, t = 0.61, P = 0.54; mutant genotype at two loci of any of the three SNPs, t = 1.16, P = 0.24). For gametocyte positivity in the population-based analysis, all three loci were significantly associated with an increase in gametocyte positivity: *rs1427407*-TT (P = 0.002), *rs11759553*-AA (P = 0.04) and *Xmn1-HBG2*-TT (P = 0.006). Gametocyte positivity increased with the number of SNPs (N = 229: mutant genotype at one locus of any of the three SNPs, t = 1.53, P = 0.13; mutant genotype at two loci of any of the three SNPs, t = 5.01, P < 0.001) and this association was maintained even upon exclusion of any individuals with HbS (N = 203, mutant genotype at one locus of any of the three SNPs, t = 1.07, P = 0.29; mutant genotype at two loci of any of the three SNPs, t = 2.16, P < 0.05) (Fig 1). Inheriting two HbF-related SNPs increased significantly gametocyte positivity as compared to inheriting only one SNP with and without inclusion of individuals with HbS (Inclusion of HbS individuals, t = 4.64, P < 0.05; exclusion of HbS individuals t = 1.79, P = 0.037).

We confirmed that the HbF-related SNPs, *rs1427407, rs11759553 and Xmn1-HBG2* (*rs7482144*), were associated with increased production of HbF. Despite variable effects of the SNPs, we also observed that the HbF-related SNPs afford some protection from clinical malaria, but increased gametocyte positivity and thus potential parasite transmissibility in two sites differing ten-fold in malaria transmission intensity.

**Fig 2:**
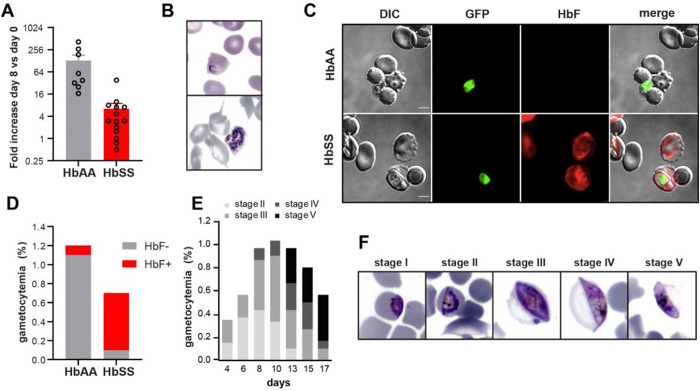
Development of *P. falciparum* in HbSS erythrocytes containing HbF. A. Increase in asexual parasitemia after 8 days of culture in HbAA or HbSS erythrocytes under an atmosphere of 1% to 2% O_2_. Circles show the number of independent experiments in blood from 8 HbAA donors and 14 HbSS donors containing HbF (from 2.1 to 21%). Parasitemia increased in blood of 11 out of 14 HbSS donors. B. Giemsa-stained images of ring-infected (top panel) or schizont-infected (bottom panel) or HbSS erythrocytes containing HbF. C, D. The presence of GFP-expressing gametocytes (green) in HbAA or HbSS erythrocytes containing 56% HbF-positive cells was analyzed by immunofluorescence analysis (C) and by flow cytometry (D). Erythrocyte staining with fetal hemoglobin monoclonal antibody highlighted that gametocytes preferentially develop in HbF-positive cells. DIC: differential interference contrast. The bars represent 2 µm. E, F. Follow-up of gametocytemia on Giemsa-stained smears show that gametocytes are able to complete their maturation in HbSS erythrocytes containing 12% HbF. Cultures were grown during 17 days under an atmosphere of 5% CO_2_, 1% O_2_ and 94% N_2_. Fig 1A: each dot represents a biological replicate. Figs 1C–1D: the experiment was done only once with the blood from one patient. Figs 1E–1F: the follow-up of gametocytemia was performed in technical triplicate with the blood from one patient containing 12% HbF.

To explore how HbF influences parasite growth, we took the extreme case of growth within HbSS erythrocytes. Early work showed that cultures of *P. falciparum* in HbSS erythrocytes under low oxygen (1–5%) exhibit rapid inhibition of growth [30,31]. The sickling of HbSS erythrocytes kills and lyses most or all of the intracellular parasites in less than 6 hours, leading to the death of all parasites in 4 days [30,32]. Here, we show that presence of HbF in HbSS erythrocytes allows the replication and development of *P. falciparum* asexual parasites during at least 8 days (Figs 2A–2B). HbF retards the polymerization of deoxy-sickle hemoglobin, the root cause of sickle cell disease, thereby enabling parasite development (Figs 2C–2D) [33]. Under these conditions, although the parasite replicates at a lower rate than in normal HbAA cells, RBCs containing HbF allow appearance and complete maturation of gametocytes (Figs 2C–2F).

## Discussion

The occurrence of several QTL that were associated with hereditary persistence of fetal hemoglobin (HPFH) in our study and found at high frequencies underlines the importance of HbF in alleviating the negative effects of hemoglobinopathies. That being said, the percentage of HbF found in individuals was relatively low at ~1.5%. Adults in the general population harbour levels of HbF at <0.6% following a rapid decrease post-birth and protective effects for modulating the negative effects of SCD require much higher levels of HbF [34,35]. Whilst we did observe a rapid decrease with age, it is however surprising that given such low levels, associations with known HbF-related SNPs were observed. One possible explanation comes from the fact that HbF levels and the occurrence of F cells, containing sufficient HbF, are not linear, although with such low levels, few if any functional F cells would be expected [34].

Despite this relatively low level of HbF, not only do we find associations of several HbF-related SNPs with the HbF levels but also our studies show that these HbF QTL contribute to protection from clinical malaria and increased gametocyte positivity. The variable strengths of the associations with protection from clinical malaria and increased gametocyte positivity in the two cohorts likely reflects their differing genetic backgrounds and the differing genotype frequencies of these HbF-related SNPs. It is reasonable to speculate that HbF is acting on these two malaria phenotypes through an impact on parasite growth. Our *in vitro* studies did show reduced parasite growth in HbSS cells in the presence of HbF, concurring with previous *in vitro* and mouse model studies demonstrating reduced parasite replication in the presence of HbF [13,36]. However, as recently reviewed, there is still uncertainty about the real impact of HbF on parasite growth [37]. The review concludes that at best the impact on growth of HbF is small and only significantly so in environments of high oxidative stress [37]. Epidemiological studies such as performed here, however, cannot unravel such effects on parasite growth without multiple consecutive timepoints from the same individual.

It must, however, be noted that HbF levels can alter following parasite infection; here we found HbF levels were 30% higher in infected individuals as compared to uninfected individuals. Induction of HbF following infection may therefore result in the observed associations of the HbF-related SNPs with the malaria phenotypes. One possible mechanism is through the dysregulated expression of the oncogene *BCL11A*, wherein lies *rs1427407* (erythroid-specific enhancer), which may influence F cell production by affecting the kinetics of erythropoiesis [11,38]. Stress erythropoiesis, which is associated with increased HbF, implicates erythropoietin in initiating downstream signaling pathways that activate *HBG* expression [39]. The *BCL11A*-associated SNP reduces its expression and disrupts binding of transcription factors crucial for switching from gamma to beta globin production [40,41]. The absence of these transcription factors enables binding of TGF-beta-induced Kruppel-like factors, KLF10 and KLF11, with subsequent increase in gamma globin production [41]. High levels of TGF-beta are associated with an anti-inflammatory response to parasite infection and hence predict a positive association of parasitic infection and HbF levels in individuals carrying the *BCL11A*-associated SNP, as observed here. Our previous genome linkage analyses on asymptomatic parasite infections in these populations suggested the locus containing *KLF11* (LOD score ≈ 2.5) (S4 Fig), consistent with the result observed here [19].

Despite the uncertainty of the root cause, the strong effect of these QTL, especially *rs1427407*, on gametocyte production is consistent with *in vitro* studies demonstrating an induction of gametocyte production in reticulocytes that were derived from HPFH and sickle cell patients [42]. Gametocyte production has been associated with the hematological environment: abnormal hemoglobin genes (S and C) and anemia induce gametocyte production *in vivo*, suggesting a response by the parasite to the prevailing hematological condition [1,4,5,43].

The high frequency of HbF QTL in sickle cell and beta-thalassemia conditions, both of which confer resistance to malaria, likely provides a strong signal for parasite sexual differentiation. Hence, whilst the individual remains protected from the severe consequences of infection, gametocyte survival in HbF RBCs will increase malaria transmission at the community level. Thus, once again the parasite has developed a remarkable capacity to respond productively to an environment hostile to its proliferation. Humans, on the other hand, have accumulated successive mutations to alleviate the negative effects of previous malaria protective mutations, but in doing so ironically enable parasite survival.

Whilst the role of human genetic determinants for the clinical malaria have been extensively studied [7,8,19,44–48], that associating with gametocyte positivity has been less well studied [2,4,5]. Many non-human genetic factors, including age, parasitaemia, anti-malarial treatment, multiplicity of infection, anemia and severity of infection to name but a few, have been associated with gametocyte positivity [49–54]. It is therefore unclear the extent to which the results observed here contribute to the overall level of parasite transmission. However, that there is a genetic determination of a blood phenotype that seemingly simultaneously reduces clinical episodes and increases gametocyte positivity does again highlight the potential impact of the malaria parasite on the human genome with unexpected consequences [44]. In conclusion, this study provides additional insight into the relationship between HbF and malaria phenotypes. Further study is clearly needed, especially on the actual infectivity of gametocytes growing in HbF rich cells and thus a true estimate of the contribution of an HbF rich environment to parasite transmission.

## Supporting information

S1 Fig Three QTLs associated with HPFH (Image from (21)). (DOCX)

S2 Fig Distribution of HbF levels in the two cohorts. (DOCX)

S3 Fig Decrease of HbF levels as a function of age in the two cohorts. (DOCX)

S4 Fig Genome scan linkage analysis results of the prevalence of asymptomatic infections in individuals from the Dielmo cohort (19). Shown is the result from Chromosome 2p and the location of the *KLF11* gene.
(DOCX)

S1 Table Genotype frequencies of selected mutations. (DOCX)

S1 File Genetic Relatedness matrices. (XLSX)

S2 File Inclusivity-in-global-research-questionnaire. (DOCX)
