# Supplementary material for: Fetal hemoglobin enables malaria parasite growth in sickle cells but augments production of transmission stage parasites
Source: PLoS One. 2025 Jul 8;20(7):e0325797. doi: 10.1371/journal.pone.0325797 (PMC12237050; doi:10.1371/journal.pone.0325797)
Supplement: S1 Fig — (DOCX) [file pone.0325797.s001.docx]

**
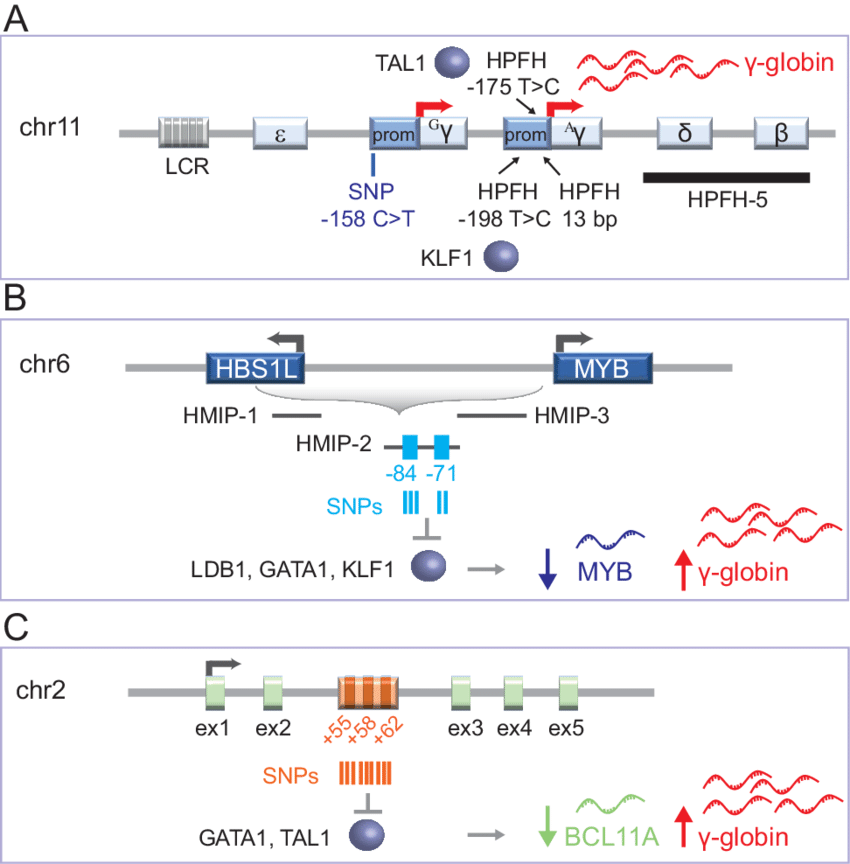
**

Xmn-1 (position -158)

MYB locus:

Myeloblastosis transcription factor

BCL11A–

zinc finger transcription factor

Oncogene B-cell lymphoma 11A
