# Supplementary figures and images for: Fetal hemoglobin enables malaria parasite growth in sickle cells but augments production of transmission stage parasites
Source: PLoS One. 2025 Jul 8;20(7):e0325797. doi: 10.1371/journal.pone.0325797 (PMC12237050; doi:10.1371/journal.pone.0325797)

**
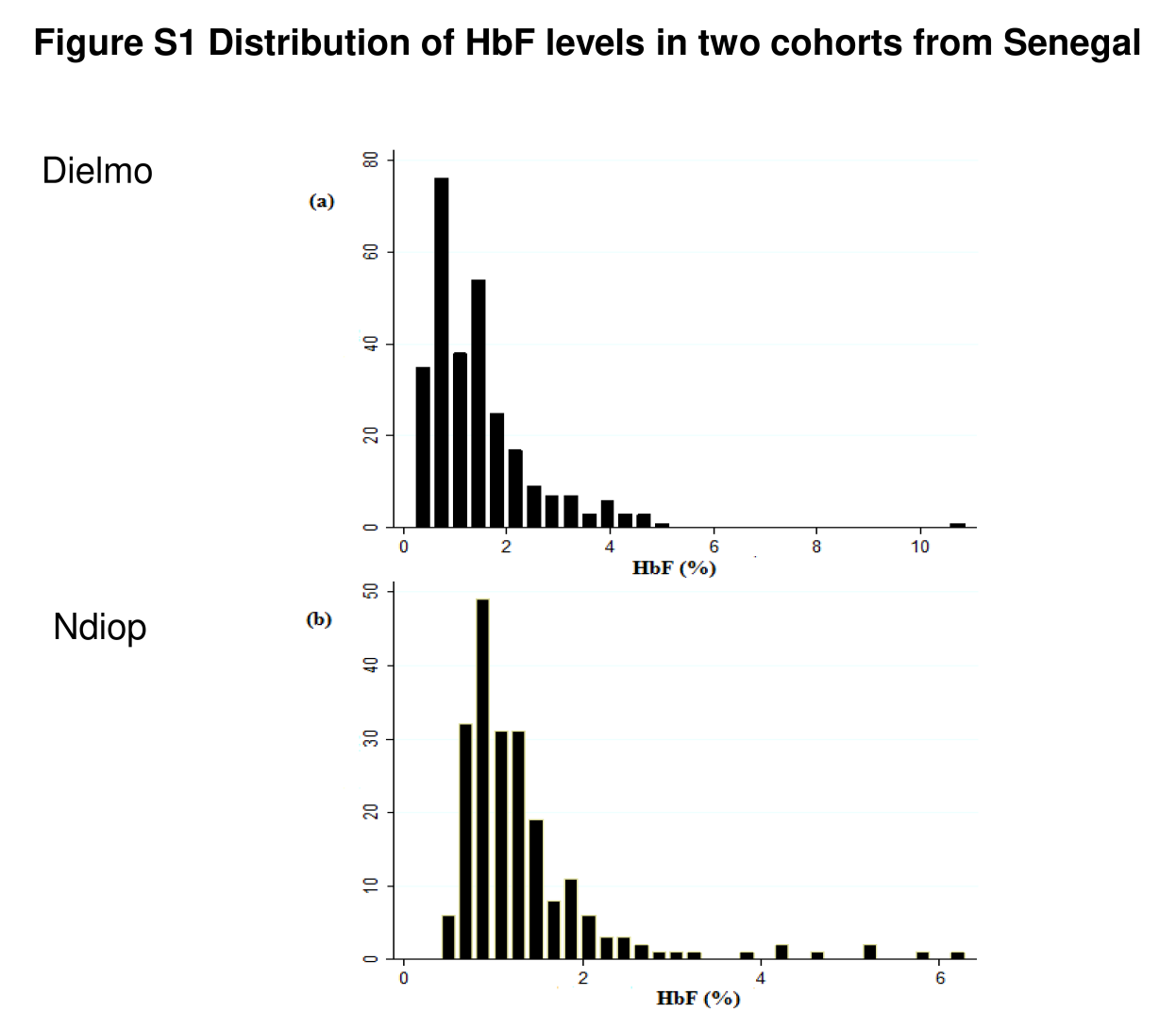
**

**Number of people**

**Number of people**

Supplement: S2 Fig — (DOCX) [file pone.0325797.s002.docx]

**A. Dielmo**

**
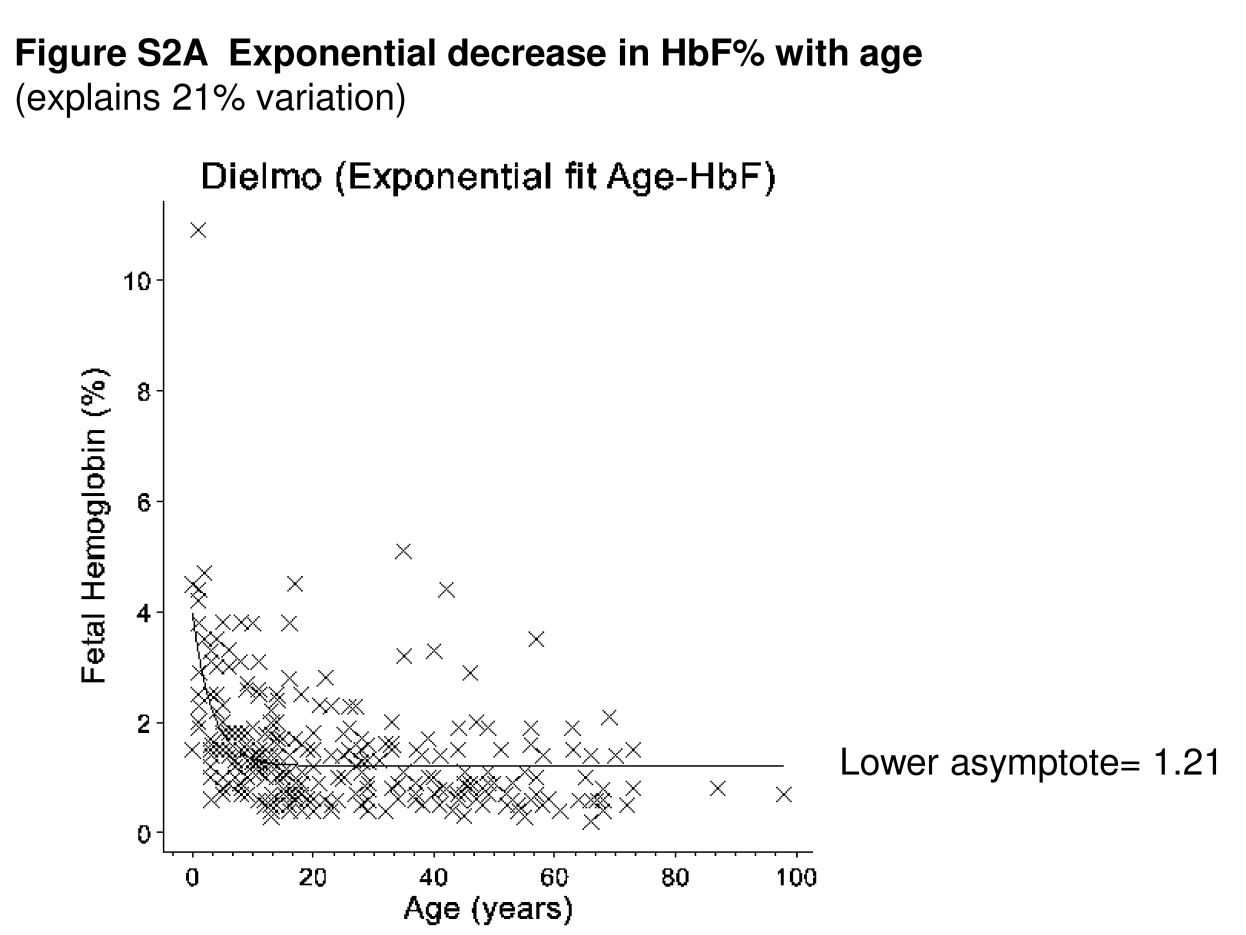
**

**B. Ndiop**

**
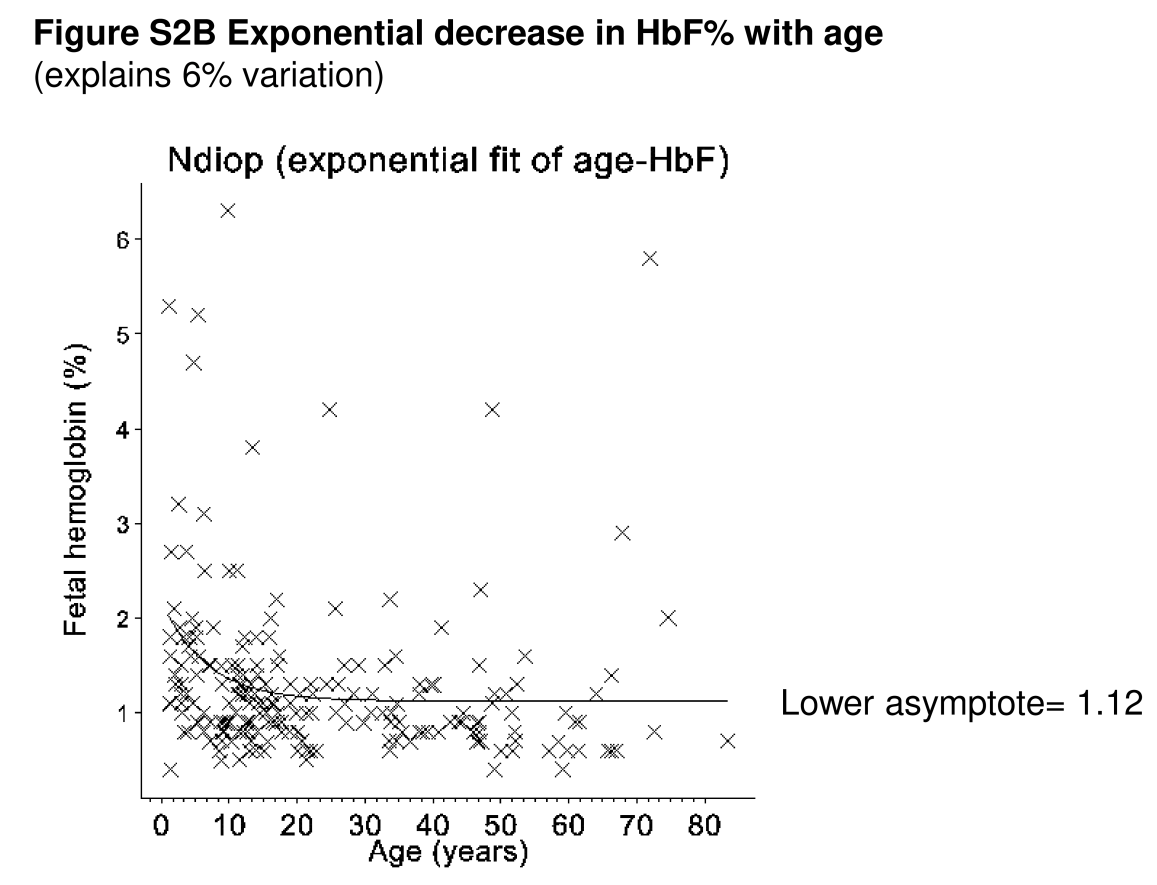
**

Supplement: S3 Fig — (DOCX) [file pone.0325797.s003.docx]

*KLF11*


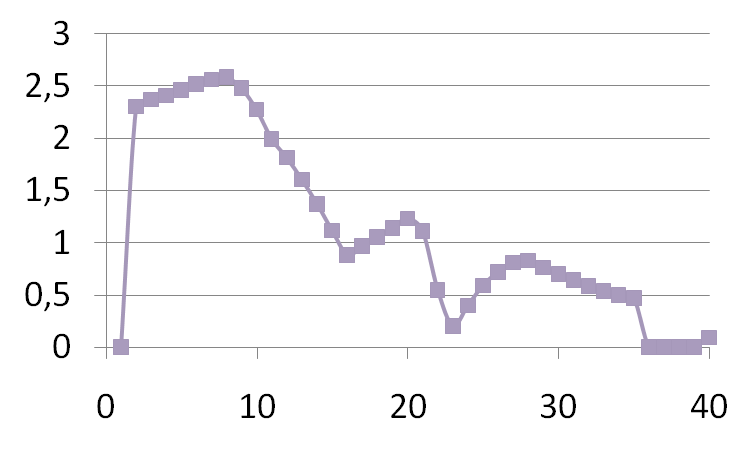


cM

LOD score

Supplement: S4 Fig — Shown is the result from Chromosome 2p and the location of the KLF11 gene. (DOCX) [file pone.0325797.s004.docx]
