# Supplementary material for: Fetal hemoglobin enables malaria parasite growth in sickle cells but augments production of transmission stage parasites
Source: PLoS One. 2025 Jul 8;20(7):e0325797. doi: 10.1371/journal.pone.0325797 (PMC12237050; doi:10.1371/journal.pone.0325797)
Supplement: S1 Table — (DOCX) [file pone.0325797.s005.docx]

| Chromosome | Position | SNP | Genotype | Dielmo | Ndiop |
| --- | --- | --- | --- | --- | --- |
| 11 | Beta-globin gene | HBB | AA | 352 | 456 |
|  |  |  | AS | 37 | 60 |
|  |  |  | SS | 2 | 0 |
|  |  |  |  |  |  |
| 11 | -158 of HBG2 gene | Xmn1 | CC | 216 | 255 |
|  |  |  | CT | 148 | 196 |
|  |  |  | TT | 20 | 31 |
|  |  |  |  |  |  |
| 2 | 60,379,872 | rs243027 | GG | 28 | 23 |
|  |  |  | GT | 120 | 134 |
|  |  |  | TT | 231 | 312 |
|  |  |  |  |  |  |
| 2 | 60,481,462 | rs6732518 | CC | 57 | 54 |
|  |  |  | CT | 196 | 215 |
|  |  |  | TT | 119 | 191 |
|  |  |  |  |  |  |
| 2 | 60,490908 | rs1427407 | GG | 168 | 239 |
|  |  |  | GT | 149 | 188 |
|  |  |  | TT | 47 | 38 |
|  |  |  |  |  |  |
| 6 | 135,061,842 | rs6904897 | GG | 46 | 38 |
|  |  |  | GT | 185 | 169 |
|  |  |  | TT | 148 | 259 |
|  |  |  |  |  |  |
| 6 | 135,097,880 | rs9399137 | CT | 16 | 31 |
|  |  |  | TT | 362 | 628 |
|  |  |  |  |  |  |
| 6 | 135,101,158 | rs11759553 | AA | 89 | 105 |
|  |  |  | AT | 185 | 220 |
|  |  |  | TT | 104 | 133 |
|  |  |  |  |  |  |
| 6 | 135,105,435 | rs4895441 | AA | 340 | 417 |
|  |  |  | AG | 36 | 94 |
|  |  |  |  |  |  |
| 6 | 135,110,502 | rs11154792 | CC | 12 | 16 |
|  |  |  | CT | 63 | 99 |
|  |  |  | TT | 253 | 319 |
|  |  |  |  |  |  |
| 6 | 135,122,074 | rs1320963 | AA | 54 | 63 |
|  |  |  | AG | 153 | 226 |
|  |  |  | GG | 127 | 165 |
